# The Associations of Parental Harsh Discipline, Adolescents’ Gender, and Grit Profiles With Aggressive Behavior Among Chinese Early Adolescents

**DOI:** 10.3389/fpsyg.2020.00323

**Published:** 2020-03-13

**Authors:** Guanyu Cui, Xiaoyu Lan

**Affiliations:** ^1^Department of Psychology, School of Education, Wenzhou University, Wenzhou, China; ^2^Faculty of Psychology, Beijing Normal University, Beijing, China; ^3^Department of Developmental Psychology and Socialization, University of Padova, Padua, Italy

**Keywords:** paternal harsh discipline, maternal harsh discipline, aggressive behavior, gender differences, grit, early adolescence

## Abstract

Although the association between parental harsh discipline and aggressive behavior in adolescence has been well established, little attention has been paid to early adolescence. Moreover, the risk and protective factors (the interplay of parents’ and adolescents’ gender, the role of grit) in this association during this period are still less explored in the literature. Guided by a socioecological framework, the current study (more exploratory in nature) identified the grit profiles based on two dimensions (i.e., perseverance and consistency) in a sample of Chinese early adolescents; likewise, this study further investigated gender-specific patterns and the moderating role of grit profiles in the association between parental harsh discipline and aggressive behavior. A total of 1,156 Chinese early adolescents (46.5% girls) were involved in this study and completed a set of self-report questionnaires. Latent profile analysis revealed three profiles of grit: low perseverance and low consistency, high perseverance and low consistency, and high perseverance and high consistency. Moreover, linear regression analysis indicated that paternal and maternal harsh discipline were each positively associated with aggressive behavior. The positive association between paternal harsh discipline and aggressive behavior was only significant for adolescent boys with low levels of perseverance and consistency; in contrast, the positive association between maternal harsh discipline and aggressive behavior was significantly stronger for adolescent boys with high levels of perseverance and consistency. These findings suggest that parental harsh discipline presents a risk factor for aggressive behavior, especially for adolescent boys in early adolescence; such a vulnerable effect is more heightened for those with low levels of perseverance and consistency. In addition, although grit is assumed to be a positive personal attribute, maternal harsh discipline to boys in the Chinese family context may disturb their positive development pathway during early adolescence, which is highly discouraged.

## Introduction

Aggression and violence remain a major social and health concern that affects large numbers of adolescents across the world (Dodge, 2008). As documented by prior research (Card and Little, 2006; Chen et al., 2010; Gangel et al., 2017), high aggressive tendencies in adolescence can lead to a series of adverse psychosocial and academic outcomes, such as internalizing problem behavior, low social competence, and poor academic achievement. Although the association between parental harsh discipline (e.g., psychological aggression, corporal punishment, and physical abuse) and aggressive behavior in Chinese adolescents has been well documented (Wang and Liu, 2014, 2018; Liu and Wang, 2018), relatively little attention has been paid to early adolescence.

As youth enter adolescence, the rate of aggressive behavior at school is seemingly booming. This is because the biological changes within the onset of puberty are troubling or disturbing to adolescents, which may intensify aggressive behavior in such a period (Tremblay, 1998; Pomerantz et al., 2017). Embedded in Confucianism, Chinese culture pays special attention to maintaining harmonious interpersonal relationships and family dignity (Bond, 2010), which has a significant influence on the development of personality, social skills, and social behavior. Expanding interpersonal relationships are more intense in early adolescence than in other developmental stages (Berndt, 2006). In this regard, aggressive behavior regarded as “highly problematic and abnormal” is culturally inhibited. Therefore, further investigation into understanding the conditions of the association between parental harsh discipline and aggressive behavior in early adolescence is essential due to the increasing aggressive tendencies in this period.

Moreover, little attention has been paid to simultaneously investigating the roles of paternal and maternal harsh discipline and the potential interplay between parents’ and adolescents’ gender in adolescents’ aggressive behavior. Along with the potential gender differences, other possible risk or protective factors (e.g., grit, perseverance, and passion for long-term goals; Duckworth et al., 2007) that may potentially influence the harsh discipline–aggression link are less investigated. It is potentially critical to further demonstrate the modifiable factors in such an association during early adolescence, given that such an investigation can inform the targeted intervention or prevention programs.

The delineation of risk and protective factors in the present study emerges from the socioecological framework (Bronfenbrenner, 1979). This framework is one of the widely used theories for understanding the multifaceted and interactive effects of personal and environmental factors that determine behaviors^1^ ; as a relational phenomenon, the development of violence and aggressive behavior emerges from interactions among individual attributes and their environment. In this regard, the understanding of aggressive behavior in early adolescence should consider not only contextual factors (e.g., parental harsh discipline) but also individual factors (e.g., adolescents’ gender and grit) that are salient in the ecology of adolescent life. Such a framework has been successfully applied to explore risk and protective factors of aggression in adolescence (Jiménez and Estévez, 2017).

To briefly summarize, the current study, more exploratory in nature, aimed to address the direct and interactive associations of paternal harsh discipline, maternal harsh discipline, adolescents’ gender, and grit with aggressive behavior in a sample of Chinese early adolescents. In the following sections, we conduct an up-to-date literature review to figure out the possible associations among study variables.

### Parental Harsh Discipline and Aggressive Behavior

As suggested by the socioecological framework (Bronfenbrenner, 1979), parenting is the most proximal factor in the study of adolescent development, such as the development of aggression and violence (Labella and Masten, 2018). This may be amplified in the context of Chinese culture, given the central role of family relations in individual networks (Chen et al., 2001; Lan et al., 2019c). According to the Chinese proverb – “to beat and scold are the emblem of love” – traditional Chinese culture tends to regard parental harsh discipline as an embodiment of parental involvement, guidance, and love (Chao, 1994). In line with these cultural influences, most Chinese parents adopt harsh discipline to motivate their children to achieve higher academic grades, socially appropriate behaviors, and positive psychological adjustment at school (Chao, 1994; Wang and Liu, 2014).

As one of the most prevalent disciplinary techniques in Chinese families (Wang and Liu, 2014), parental harsh discipline involves parenting with higher levels of power assertion, as well as parenting with behavioral and psychological control, psychological aggression, corporal punishment, and severe physical abuse (Jansen et al., 2012; Wang and Liu, 2014). When parents use harsh discipline to behaviorally and psychologically control their children’s behavior, their children may learn the effectiveness of hostile and manipulative behavior via learning processes (Bandura, 1978), which could generalize to aggression in other social interaction contexts (Meter et al., 2019). Such a theoretical assertion has been demonstrated in several lines of empirical research. For instance, Chen et al. (2001) found that maternal and paternal power assertion are positively associated with aggression in Chinese children. Similarly, research has shown that harsh parenting is positively related to proactive and reactive aggression (Xu et al., 2009), indicating that parental harsh discipline results in higher levels of aggressive behavior in Chinese adolescents.

Despite these well-established associations, some disciplinary gaps still exist. The majority of research has centered on the role of mothers in influencing adolescents’ adjustment and often regarded the fathers’ role as indirect in comparison with the direct function of mothers. In response to this research gap, a burgeoning body of research has indicated the indispensable role of fathers in adolescents’ behavioral development (Lamb, 2004; Li and Lamb, 2013; Pace et al., 2018, 2018a,b). For Chinese adolescents, Shek (2007) has found that fathers and mothers adopt different but mutually complementary parenting styles, such as “kind mother, strict father” or “strict mother, kind father.” Given that fathers and mothers may play different roles in educating their children (Shek, 2007; Jansen et al., 2012; Wang and Liu, 2014), little attention has been paid to simultaneously investigating the roles of paternal and maternal harsh discipline in adolescents’ aggressive behavior. Attempting to fill this gap, the current study simultaneously examined the roles of paternal and maternal harsh discipline in adolescents’ aggressive behavior.

### The Interplay of Parents’ and Adolescents’ Gender

Extant research incorporating parents’ or adolescents’ gender has investigated whether and how parental harsh discipline is linked to adolescents’ psychosocial functions (e.g., Wang and Kenny, 2014). However, to our knowledge, less is explored in terms of the possibility that the harsh discipline–aggression link may differ depending on both parents’ and adolescents’ gender. It would be interesting and valuable to explore this striking gap within the Chinese context due to the following considerations.

First, gender differences exist in aggressive behavior (Björkqvist, 2018). Typically, boys tend to show more inappropriate or uncontrollable behaviors than girls in daily life. Among boys, gender socialization is regarded as a variable linked to the tendency to display aggressive behavior. For instance, they are more prone to achieving manhood through a process of distancing femininity from themselves and maintaining a hierarchy of social superiority of masculinity (D’Urso and Pace, 2019). Second, influenced by traditional Chinese culture, gender-differentiated parenting still strongly impacts Chinese adolescent’s socialization (Ngai et al., 2018; Xing et al., 2019). For instance, Xing et al. (2019) have demonstrated that parents are more likely to use strict strategies to discipline boys even when they exhibit the same misbehaviors as girls. Third, Chinese fathers often take more responsibility in disciplining their sons but leave the parenting of daughters to mothers (Ho, 1986); that is, adolescents usually spend more time with their same-gender parent. Thus, it is conceivable that parents have more opportunities to discipline their same-gender children, and parental harsh discipline may be more likely to trigger misbehaviors for same-gender adolescents (Xing et al., 2019).

Apart from gender differences, Chen et al. (2001) found that maternal warmth is negatively related to aggression mainly for compliant Chinese children and that paternal warmth is conversely associated with aggression for non-compliant and defiant Chinese children. This indicates that other personal attributes may moderate the interplay of parents’ and adolescents’ gender in aggression. Therefore, the present study also explored whether the interaction between parents’ and adolescents’ gender in aggressive behavior was moderated by individual characteristics, such as grit, among Chinese early adolescents.

### The Moderating Role of Grit

Grit refers to perseverance and passion for long-term goals, especially in the face of difficulties and adversities (Duckworth et al., 2007). To date, grit has been predominantly studied in the context of achievement and performance outcomes (Credé et al., 2017; Datu et al., 2017b). Although some researchers have examined the mediation models in which grit mediates the association between personal/environmental factors and academic outcomes (e.g., Howard et al., 2019; Tang et al., 2019), less work has explored whether parental harsh discipline interacts with grit in explaining the variance of aggression. From a theoretical perspective, the development of aggressive behavior emerges from interactions among individual attributes and their environment (Bronfenbrenner, 1979; Jiménez and Estévez, 2017). Such an approach in the grit literature has been documented in many recent findings (e.g., Lan et al., 2019a; Lan and Radin, 2019). Statistically, given the nature of cross-sectional research design, conducting mediation analyses is not recommended, as it yields inherently misleading results (Maxwell and Cole, 2007). Considering these theoretical and statistical perspectives, we adopted a moderation model (i.e., interactive effect), rather than a mediation model (i.e., indirect effect), in the present study to address the research question.

Recently, an emerging body of research has also indicated the positive association of grit with psychosocial outcomes, especially under unfavorable conditions. For example, high levels of grit are found to buffer the relationship between adverse life events and suicidal ideation (Blalock et al., 2015). As for Chinese early adolescents, Lan and Moscardino (2019) found that, in the context of negative teacher–student relations, high levels of grit can promote Chinese early adolescents’ school satisfaction and learning engagement. Indeed, highly embedded in Confucianism, Chinese culture emphasizes diligence and perseverance when facing setbacks and challenges (Lan et al., 2019a). Given these empirical findings and cultural characteristics, we proposed that grit may buffer against the association between parental harsh discipline and aggressive behavior in Chinese early adolescents.

Notwithstanding intriguing study findings of overall grit, the two grit facets have demonstrated unique validity for performance outcomes (Credé et al., 2017). Specifically, grit comprises two aspects: perseverance of effort (hereafter, “perseverance”) and consistency of interests (hereafter, “consistency”). Higher levels of perseverance enable individuals to endure challenges and difficulties through sustaining personal efforts and determination to achieve long-term ambitions. In contrast, consistency attaches importance to attaining long-term aspirations by centering on their own interests and targeted tasks (Duckworth and Quinn, 2009). A meta-analytic synthesis of the grit literature further indicates significant effect size differences between the two facets in predicting achievement, retention, and intelligence outcomes (Credé et al., 2017), suggesting that the potential utility of studying each facet separately is more informative and valuable. Such an indication has been recently applied to several empirical studies (e.g., Disabato et al., 2019; Lan and Radin, 2019). For instance, Lan and Radin (2019) found that high levels of perseverance buffer the association between peer attachment and problem behaviors in migrant adolescents, but the corresponding association is not significant in non-migrant adolescents. In contrast, high levels of consistency buffer the association between peer attachment and problem behavior for both migrant and non-migrant adolescents. This finding may indicate that perseverance is more pronounced in adolescents under the vulnerable context, whereas consistency is a general attribute that applies to all individuals. Similarly, research conducted in a collectivist culture has demonstrated that perseverance is more salient than consistency in predicting key psychosocial and academic outcomes (Datu et al., 2016). Overall, these findings consistently suggest that grit researchers should explore separate results for perseverance and consistency due to substantial prediction differences between the two facets (Disabato et al., 2019).

### A Person-Centered Approach

Despite such evidence, numerous studies building on a variable-centered approach make it difficult to examine the distinct combinations of perseverance and consistency with aggressive behavior.^2^ Building on the assumption that the population is homogeneous, a variable-centered approach is valuable in terms of describing associations between study variables (i.e., concerning the relative contributions that predictor variables make to an outcome). Such an approach has gained much research attention in developmental science. Although it is informative, this approach fails to consider that the population is heterogeneous concerning how the predictors operate on the outcome.

To balance the disproportion of the existing literature (mostly focusing on a traditional variable-centered approach), the current study adopted a person-centered approach to identify potential grit profiles (our research focus is on the individual and not on the variable). A person-centered approach can identify relatively homogeneous groups of adolescents with distinct configurations in terms of individuals’ characteristics, such as perseverance and consistency. We assumed that adopting a person-centered approach to exploring the moderating role of grit in the association between parental harsh discipline and aggressive behavior is essential, in order to examine how specific grit profiles may relate to behavioral outcomes. Moreover, a person-centered approach involves empirically identifying patterns of a particular construct and its association with different outcomes, which is advantageous for crafting interventions that are relevant to individuals with diverse personal characteristics (Datu and Fong, 2018). In line with this merit, the current investigation may have distinct implications for Chinese adolescents when characterizing a particular group of individuals belonging to a specific grit profile.

Furthermore, adopting a person-centered approach is in line with our theoretical framework; that is, a fundamental principle in the development of the integrated individual is functional interaction (Bronfenbrenner, 1979). Individual developmental change processes get their characteristic features and properties from the functional interaction among the operating elements involved, not by the effect of each part separately (Bergman et al., 2006). Based on the available research, Datu and Fong (2018) have highlighted the importance of using a person-centered approach in assessing the role of grit constructs in different learning-related emotions. Their research exhibits three profiles: high perseverance and high consistency, high perseverance and low consistency, and low perseverance and high consistency. Chinese adolescents who belong to the profile of high perseverance and low consistency show the highest scores on hope and lowest scores on anxiety and shame. Building on their finding, we further investigated the association of grit profiles with aggressive behavior, which goes beyond academic outcomes.

### The Present Study

To summarize, the aims of this exploratory study were twofold. First, we aimed to identify grit profiles in a sample of Chinese early adolescents based on two dimensions (perseverance and consistency). Second, we strived to examine whether adolescents’ gender and grit profiles may moderate the association between parental harsh discipline and aggressive behavior.

Given the relative scarcity of research on this issue, we did not generate *a priori* hypotheses regarding study associations. Instead, we relied on an exploratory rather than confirmatory approach, based on the assumption that aggressive behavior is a complex process that involves various individual and contextual components. In this context, it is hardly captured in a single confirmatory model (Jiménez and Estévez, 2017). This exploratory approach makes it possible to simultaneously consider all variables that potentially contribute to understanding the structure of the data.

Despite the exploratory nature of the current study, according to the aforementioned theoretical, empirical, and cultural perspectives, some exploratory expectations may be present. First, we expected three grit profiles: high perseverance and high consistency, high perseverance and low consistency, and low perseverance and high consistency. Second, we assumed that early adolescents with higher levels of paternal and/or maternal harsh discipline would report higher levels of aggressive behavior (compared with those adolescents reporting lower levels of parental harsh discipline). Moreover, paternal and maternal harsh discipline may be more likely to trigger high levels of aggressive behavior for their same-gender children. In addition, the grit profile, highlighting high perseverance, may buffer against these associations. Specifically, for boys with high perseverance, paternal harsh discipline and aggressive behavior are less strong than for those reporting low perseverance; for girls with high perseverance, maternal harsh discipline and aggressive behavior are less strong than for those reporting low perseverance.

## Materials and Methods

### Participants and Procedures

The current study was based on the project entitled “Chinese Early Adolescents’ Psychosocial and Academic Adjustment at School.” Prior to data collection, ethical approval was obtained from the affiliated university and schools. The corresponding author contacted several public elementary schools located in north mainland China (Harbin, Lanzhou, and Beijing). After getting the approval from school principals, an information brochure and informed consent were sent to each adolescent at school, who was asked to bring them home for his or her parents’ approval. In the meantime, each adolescent was asked whether he or she was willing to participate in this project. Overall, the participation rate was 93%, which is in line with prior research of Chinese adolescents (Lan et al., 2019b).

Data collection was conducted in October 2017 (the middle of a semester), with the assistance of several research assistants and postgraduate students. During school hours, research assistants administered the instruments and provided a standardized set of instructions to ensure that information regarding participant rights was communicated uniformly across classrooms. These rights included the option to withdraw and debrief in addition to the anonymity and confidentiality of their participation. Adolescents were asked to complete these questionnaires in each classroom during a regular class hour.

A total of 1,156 early adolescents (46.5% girls) aged between 10 and 13 years (*M*_age_ = 10.94; *SD* = 0.81) were recruited in this study. During data collection, participants were attending fourth and fifth grades in five public primary schools (25 classes; the number of students per classroom was approximately 20–40). Sixth graders were excluded from this study because they were highly engaged in preparing for the entrance examination to middle school. With regard to parental education, 24.2, 38.8, 20.3, and 16.7% of fathers had completed middle school or lower, high school, undergraduate education, and postgraduate education or higher, respectively; 28.9, 36, 22.9, and 12.2% of mothers had completed middle school or lower, high school, undergraduate education, and postgraduate education or higher, respectively. In terms of parental occupation, most fathers (22.9%) worked as a civil servant or company employee, and most mothers (33.2%) worked in the field of social service. Based on a recent report released by the National Bureau of Statistics (2018), their family monthly incomes were moderate (800–1,200 US dollars). Additionally, most adolescents (93.1%) belonged to the Han ethnic group, which is the major ethnic group in China, to balance potential multiethnic impacts.

### Measures

#### Sociodemographic Characteristics

Sociodemographic information collected from the participants included gender, age, ethnicity, parental educational level, parental occupation, and family monthly income. Socioeconomic indicators were assessed via three indicators (i.e., parental educational level, occupation, and family monthly income; Lan and Zhang, 2019). Concerning educational level, four options were available: (a) middle school graduate or lower, (b) high school graduate, (c) bachelor’s degree graduate, and (d) master’s degree graduate or higher. Parental occupation and monthly family income were coded by different options based on occupational classification and residents’ income criteria in China (1 = unemployed or temporary work, 2 = manufacturing or service, 3 = office work, 4 = administrative or managerial, 5 = professional and technical; 1 = relying on government relief, 2 ≤ 3,000 RMB, 3 = 3,000–5,000 RMB, 4 = 5,000–8,000 RMB, 5 = 8,000–12,000 RMB, 6 = 12,000–20,000 RMB, 7 ≥ 20,000 RMB; Wang et al., 2019). As suggested by prior research (Lan and Zhang, 2019), these three scores were standardized and subsequently summed to yield an overall socioeconomic status (SES) score.

### Parental Harsh Discipline

Paternal and maternal harsh discipline was assessed by the Chinese adaption of the Ghent Parental Behavior Scale (GPBS; Van Leeuwen and Vermulst, 2004; Zhang et al., 2011). Parental harsh discipline is one of the subscales of the GPBS, and this subscale consists of 18 items (nine items for the father and nine items for the mother). One example is “When I do not obey the rules, my father/mother will spank me.” Participants were asked to assess paternal and maternal harsh discipline separately based on a five-point Likert scale ranging from 1 (*not at all like my father or mother*) to 5 (*very much like my father or mother*). The average score was yielded to represent the score of paternal and maternal harsh discipline, respectively. A higher score related to each dimension indicated higher levels of perception of harsh discipline from the father and mother. Previous research has demonstrated good internal consistency of this scale in Chinese adolescents (Li et al., 2014). Furthermore, confirmatory factor analysis (CFA) was used to ensure the construct validity (i.e., one-factor structure per each dimension) of this scale, and results showed an acceptable model fit: χ^2^(27) = 121.31, *p* < 0.001, Tucker Lewis Index (TLI) = 0.97, comparative fit index (CFI) = 0.97, standardized root mean square residual (SRMR) < 0.05 for paternal harsh discipline; χ^2^(27) = 84.08, *p* < 0.001, TLI = 0.98, CFI = 0.98, SRMR < 0.05 for maternal harsh discipline. The items also showed proper factor loadings (β = 0.46–0.66 for paternal harsh discipline, β = 0.50–0.68 for maternal harsh discipline). Additionally, internal consistency coefficients (Cronbach’s α = 0.88 for paternal harsh discipline and 0.87 for maternal harsh discipline) further supported that this scale was appropriate for the assessment of Chinese early adolescents.

### Grit

Grit was measured by the eight-item Grit Scale (Duckworth and Quinn, 2009), which has been validated in Chinese adolescents by Li et al. (2018). This scale consists of two dimensions: perseverance and consistency. Examples are “Setbacks do not discourage me (perseverance, four items)” and “New ideas and projects sometimes distract me from previous ones (consistency, four items).” Participants were asked to rate each item ranging from 1 (*not like me at all*) to 5 (*very much like me*) on a Likert scale. The average score of these two facets was calculated separately, with higher scores indicating higher levels of perseverance and consistency. A previous study showed good internal consistency of this scale in Chinese adolescents (Lan, 2019; Lan and Moscardino, 2019). Moreover, we used CFA to test a two-factor structure of grit. The results of CFA [χ^2^(19) = 135, *p* < 0.001, TLI = 0.93, CFI = 0.95, SRMR < 0.05; factor loadings ranged from 0.74 to 0.83] and internal consistency coefficients of this scale (Cronbach’s α = 0.78 and 0.79 for perseverance and consistency, respectively) were adequate.

### Aggression Behavior

Aggressive behavior was measured via a subscale of the Youth Self-Report (YSR; Achenbach, 1991), which is derived from the Child Behavior Checklist (CBCL) for the purpose of a self-report. YSR has been validated in Chinese adolescents (Leung et al., 2006), showing adequate reliability and validity. This subscale consists of nine items, and one of the examples is “I destroy my own or others’ belongings.” Participants were asked to rate each item from 1 (*definitely does not apply to me*) to 4 (*definitely applies to me*) on a Likert-type scale. The average score of all the items was calculated, with a higher score indicating severe aggressive behavior. A previous study showed good internal consistency of this scale in Chinese adolescents (Lan et al., 2019a). In the current study, the results of CFA [i.e., one-factor structure; χ^2^(27) = 342.74, *p* < 0.001, TLI = 0.90, CFI = 0.93, SRMR < 0.05; factor loadings ranged from 0.44 to 0.59] and the internal consistency coefficient (Cronbach’s α = 0.89) indicated that this scale was adequate in the measurement of Chinese early adolescents.

### Analytical Plan

Data analyses were performed using R software (R Core Team, 2017) and Mplus 7.0 (Muthén and Muthén, 2012). Preliminary exploratory analyses (e.g., scatterplot, Q–Q plot, and correlation matrix) were conducted to examine whether the data met the assumptions of linear regression and the presence of potential outliers in this study. The graphs and correlation matrix showed that the data fit the premises, and no extreme outliers were identified (Lan et al., 2019d). Moreover, 20 cases were excluded due to high rates of missing data (>20%) in at least one of the questionnaires in our battery. To investigate the impact of missing data (<20%), a Little’s Missing Completely at Random (MCAR) test was performed (Lan and Wang, 2019). Results supported the MCAR assumption, χ^2^(433) = 458.14, *p* = 0.20, and thus, full-information maximum-likelihood estimates were employed to impute missing data.

Before addressing our research expectations, we computed means, standard deviations, and zero-order correlations to have a preliminary overview of study variables. In response to the first research aim (i.e., exploring grit profiles), a latent profile analysis was implemented in Mplus 8.0 (Muthén and Muthén, 2012). We decided to use latent profile analysis instead of cluster analysis in the present research, given that the former is preferable to the latter when there is a potential significant correlation among indicators. This is because multicollinearity may skew the cluster analysis results toward those variables that are highly correlated. In contrast, latent profile analysis relies on probabilities and fit statistics to identify the optimal number and nature of subgroups, and thus, multicollinearity and standardization are less of a concern (Stanley et al., 2017). Moreover, using a relatively large sample size in the present study could potentially increase the accuracy and certainty of the grit profiles (Wurpts and Geiser, 2014).

Suggested by prior research (Ma et al., 2019), one- to five-profile solutions were evaluated and compared based on fit statistics and interpretability to determine the optimal number of latent profiles. We followed the recommendations of Marsh et al. (2009) to decide the optimal model fit. That is, an optimal model fit was selected in the context of lower Akaike information criterion (AIC) values, Bayesian information criterion (BIC) values, and adjusted Bayesian information criterion (aBIC) values, as well as higher entropy, a significant bootstrapped likelihood ratio test (BLRT), and a significant Lo–Mendell–Rubin adjusted likelihood ratio test (LMR-LRT). Moreover, to determine whether potential grit profiles significantly differed on perseverance and consistency, multivariate analysis of variance (MANOVA) and a follow-up *post hoc* test (i.e., Bonferroni correction) was conducted. This was done to determine whether the natural configurations of this profile could reflect the well-defined and differentiated patterns (e.g., Muthén and Muthén, 2012; Datu and Fong, 2018).

Second, concerning the second research aim (i.e., the gender-specific patterns and the moderating role of grit profiles in the association between parental harsh discipline and aggressive behavior), a linear regression analysis was conducted in R software (R Core Team, 2017). Follow-up simple slope analyses were used to interpret the significant interaction effect (Aiken and West, 1991). Moreover, as suggested by prior research about the potential link between sociodemographic variables (i.e., age and SES) and aggressive behavior (Piotrowska et al., 2015), the current study treated age and SES as potential covariates included in the linear regression.

## Results

### Descriptive Statistics

Means and standard deviations for study variables and bivariate correlations are reported in Table 1. Paternal harsh discipline and maternal harsh discipline were each positively associated with aggressive behavior; two facets of grit were each negatively associated with aggressive behavior; boys reported higher levels of aggressive behavior than girls. In terms of covariates, SES was negatively related to aggressive behavior. No other significant correlation was found.

**TABLE 1:** Descriptive statistics and bivariate correlations of study variables for Chinese early adolescents.

	*M*	SD	Range	1	2	3	4	5	6	7	8
1. Paternal harsh discipline	2.03	0.94	1–5	–							
2. Maternal harsh discipline	1.92	0.92	1–5	0.63***	–						
3. Perseverance	4.03	0.82	1–5	−0.12***	−0.15***	–					
4. Consistency	3.40	1.03	1–5	−0.22***	−0.19***	0.20***	–				
5. Gender^a^	–	–	1–2	−0.21***	−0.11***	0.09**	0.13***	–			
6. Aggressive behavior	1.40	0.45	1–4	0.36***	0.34***	−0.23***	−0.22***	−0.21***	–		
7. Age	10.94	0.81	10–13	0.02	0.01	−0.06*	0.01	−0.06*	0.04	–	
8. Socioeconomic status	0.001	4.14	−8.19–8.29	−0.13***	−0.10***	0.11**	0.01	0.07*	−0.14***	–0.01	–

N = 1,156. ^a^Coded as 1 = male, 2 = female. *p < 0.05, **p < 0.01, ***p < 0.001.

A preliminary analysis indicated that adolescents reported higher levels of paternal harsh discipline than maternal harsh discipline, *t* = 4.48, *p* < 0.001.

### Identification of Grit Profiles

Although there is diverse opinion on how to choose the optimal number of profiles, it is useful to explore solutions with varying numbers of profiles and to select one that makes the most sense in relation to the interpretation of the results, previous research, as well as goodness-of-fit indices and tests of statistical significance (Marsh et al., 2009). The model fit indices of the latent profile analysis are reported in Table 2.

**TABLE 2:** The goodness-of-fit indices for different latent profile analysis models.

	AIC	BIC	aBIC	Entropy	LMR-LRT	BLRT	Smallest profiles (%)
1-Profile	6,184.44	6,204.65	6,191.95	–	–	–	–
2-Profile	6,070.43	6,105.80	6,083.56	0.61	114.60***	120.02***	22.0
**3-Profile**	**6,011.54**	**6,062.07**	**6,030.30**	**0.70**	61.96**	64.89**	**17.8**
4-Profile	5,896.77	5,962.46	5,921.16	0.74	115.32	120.77	10.3
5-Profile	5,704.62	5,785.46	5,734.64	0.86	189.21***	198.15***	3.89

N = 1,156. AIC, Akaike information criterion; BIC, Bayesian information criterion; aBIC, adjusted Bayesian information criterion; LMR-LRT, Lo–Mendell–Rubin adjusted likelihood ratio test; BLRT, bootstrapped likelihood ratio test. The best model fit has been set in bold. **p < 0.01, ***p < 0.001.

As shown in Table 2, the LMR-LRT and BLRT values were significant for the two-, three-, and five-profile solutions. However, the smallest profiles in the five-profile solution only accounted for 3.89% of participants. As suggested by prior research (Marsh et al., 2009), solutions with small numbers of participants (e.g., 5% of the total sample) may not truly represent a unique latent subgroup. Thus, the five-profile solution should be excluded. Recent findings have also demonstrated such an implication. For example, Wurpts and Geiser (2014), using a sample of 70 individuals, found that a profile with a relatively small sample size is not feasible under virtually any condition they examined: either there are too many convergence problems, or the profile assignment accuracy is too low to interpret the profile with this sample size. Among the remaining solutions, three-profile showed lower levels of AIC, BIC, aBIC, as well as higher entropy, compared with a two-profile solution. Taken together, we adopted a three-profile solution in the further course of analyses.

Moreover, in the current study, we decided to use a three-profile solution due to additional considerations. First, we were interested in exploring the moderating role of grit profiles in the association between parental harsh discipline and aggressive behavior in early adolescence. In this sense, if we adopted a five-profile solution, 22 interaction terms would be included in the regression analysis. For ease of the interpretation of the results, a five-profile solution was excluded. Second, such a solution (i.e., three-profile solution) is also suggested by prior empirical research on Chinese adolescents (Datu and Fong, 2018).

**FIGURE 1:**
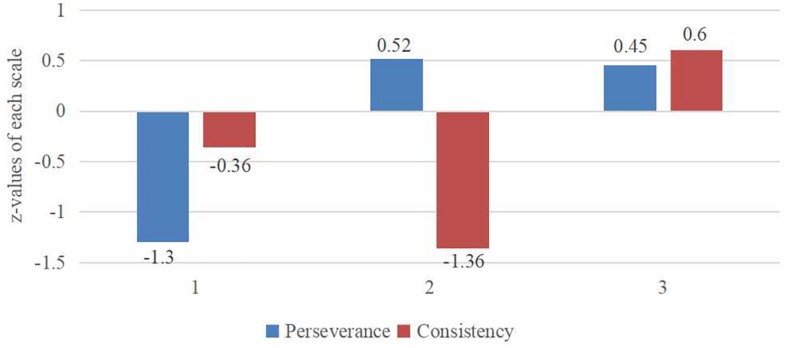
Three grit profiles based on perseverance and consistency (*z*-standardized means). *N* = 1,156.

These three profiles were interpreted based on prior research on grit profiles (Datu and Fong, 2018). The results showed that these profiles involved: (a) profile 1 (low perseverance and low consistency) = 305 early adolescents (26.4%); (b) profile 2 (high perseverance and low consistency) = 206 early adolescents (17.8%); and (c) profile 3 (high perseverance and high consistency) = 645 early adolescents (55.8%). Figure 1 visualizes the distribution of these three profiles.

In addition, MANOVA showed that these three profiles showed significant differences in perseverance [*F*(2,1,153) = 908.61, *p* < 0.001, $\eta_{\text{p}}^{2}$ = 0.61] and consistency [*F*(2,1,153) = 781.79, *p* < 0.001, $\eta_{\text{p}}^{2}$ = 0.58]. In particular, the Bonferroni test showed that significant differences were found for all possible pair-wise comparisons except for the non-significant difference in perseverance between profile 2 and profile 3.

### Associations of Parental Harsh Discipline, Adolescents’ Gender, and Grit Profiles With Aggressive Behavior

In this step of the analysis, three profiles of grit were coded into two dummy variables, and profile 1 (low on both perseverance and consistency) was treated as a reference group. This is because prior research has consistently documented that low levels of perseverance and consistency are related to adverse psychosocial outcomes (Blalock et al., 2015; Lan et al., 2019a). The results are reported in Table 3.

**TABLE 3:** Regression analysis predicting aggressive behavior from paternal and maternal harsh discipline, grit profiles, and adolescents’ gender.

Variable	*B*	*SE*	95% CI		*t*	*p*
Paternal harsh discipline (PHD)	0.07	0.02	0.03	0.11	3.38	<0.001
Maternal harsh discipline (MHD)	0.08	0.02	0.05	0.12	4.42	<0.001
Profile 2	–0.03	0.04	–0.11	0.06	–0.62	0.54
Profile 3	–0.10	0.03	–0.16	–0.04	–3.29	<0.001
Gender^a^	–0.15	0.03	–0.21	–0.09	–5.12	<0.001
Age	–0.01	0.02	–0.03	0.03	0.27	0.79
Socioeconomic status	–0.01	0.00	–0.01	0.00	–2.54	0.01
PHD × profile 2	–0.01	0.06	–0.11	0.10	–0.11	0.91
MHD × profile 2	–0.02	0.05	–0.12	0.08	–0.43	0.67
PHD × profile 3	–0.05	0.04	–0.13	0.04	–1.11	0.27
MHD × profile 3	0.07	0.04	–0.01	0.15	1.74	0.08
PHD × gender	–0.07	0.04	–0.15	0.01	–1.62	0.11
MHD × gender	–0.07	0.04	–0.15	0.01	–1.93	0.06
Profile 2 × gender	–0.01	0.08	–0.17	0.15	–0.16	0.88
Profile 3 × gender	–0.03	0.06	–0.15	0.10	–0.44	0.66
PHD × profile 2 × gender	0.06	0.11	–0.16	0.27	0.51	0.61
MHD × profile 2 × gender	0.05	0.10	–0.15	0.24	0.46	0.64
PHD × profile 3 × gender	0.20	0.09	0.03	0.37	2.34	0.02
MHD × profile 3 × gender	–0.22	0.08	–0.38	–0.06	–2.64	0.01

*N = 1,156. ^a^Coded as 1 = male, 2 = female. Profile 1 (low perseverance and low consistency) was considered as the reference group, profile 2 = high perseverance and low consistency, and profile 3 = high perseverance and high consistency. Adjusted R^2^ = 0.19. CI = confidence interval.*

As shown in Table 3, paternal and maternal harsh discipline were each positively associated with aggressive behavior; adolescents of profile 3 reported lower levels of aggressive behavior than those of profile 1; boys reported higher levels of aggressive behavior than girls; SES was negatively associated with aggressive behavior. Furthermore, the interaction of paternal harsh discipline, profile 3, and adolescents’ gender was positively related to aggressive behavior; the interaction of maternal harsh discipline, profile 3, and adolescents’ gender was negatively associated with aggressive behavior.

**FIGURE 2:**
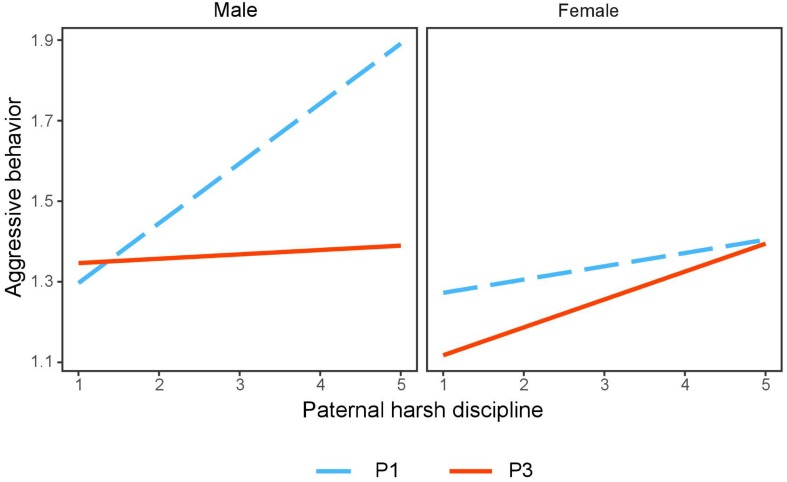
Interaction effect of paternal harsh discipline, grit profiles, and adolescents’ gender on aggressive behavior. P1, profile 1 (low perseverance and low consistency); P3, profile 3 (high perseverance and high consistency).

Follow-up simple slope analysis revealed that the positive association between paternal harsh discipline and aggressive behavior was significant for adolescent boys of profile 1 (*B* = 0.16, *SE* = 0.03, *t* = 4.56, *p* < 0.001) but not for adolescent boys of profile 3 (*B* = 0.02, *SE* = 0.04, *t* = 0.52, *p* = 0.60). By contrast, the association between paternal harsh discipline and aggressive behavior was not significant for adolescent girls from both profile 3 (*B* = 0.07, *SE* = 0.04, *t* = 1.77, *p* = 0.08) and profile 1 (*B* = 0.03, *SE* = 0.05, *t* = 0.67, *p* = 0.50; see Figure 2).

**FIGURE 3:**
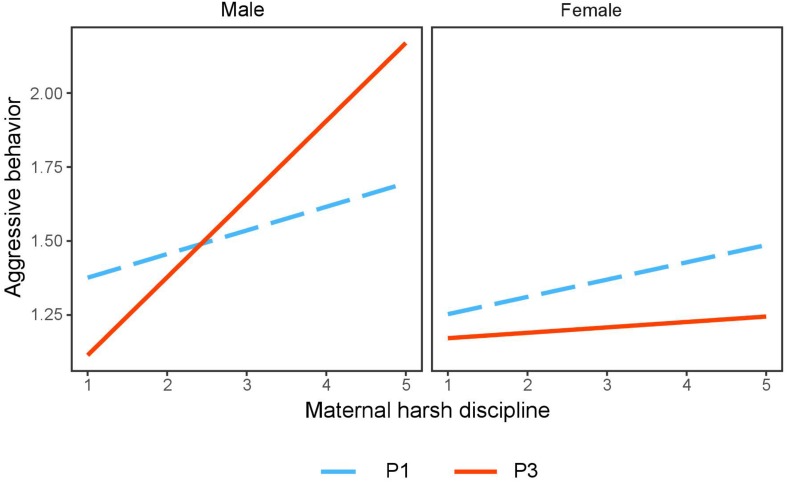
Interaction effect of maternal harsh discipline, grit profiles, and adolescents’ gender on aggressive behavior. P1, profile 1 (low perseverance and low consistency); P3, profile 3 (high perseverance and high consistency).

Furthermore, the positive association between maternal harsh discipline and aggressive behavior was significantly stronger for adolescent boys of profile 3 (*B* = 0.25, *SE* = 0.04, *t* = 6.13, *p* < 0.001) than adolescent boys of profile 1 (*B* = 0.07, *SE* = 0.03, *t* = 2.03, *p* = 0.04). In contrast, the association between maternal harsh discipline and aggressive behavior was not significant for adolescent girls from both profile 3 (*B* = 0.02, *SE* = 0.04, *t* = 0.55, *p* = 0.58) and profile 1 (*B* = 0.06, *SE* = 0.04, *t* = 1.55, *p* = 0.12; Figure 3).

## Discussion

Although abundant research suggests that parental harsh discipline detrimentally affects adolescents’ development, including via manifestations of aggressive behavior in Chinese adolescents, little is known about such an association in early adolescence. Moreover, the risk and protective factors in this association are still poorly understood. Attempting to fill these knowledge gaps, this exploratory study identified grit profiles based on perseverance and consistency in a sample of Chinese early adolescents; likewise, this study investigated the gender-specific patterns and the moderating role of grit profiles in the association between parental harsh discipline and aggressive behavior. The findings showed three empirically based grit profiles: low perseverance and low consistency, high perseverance and low consistency, and high perseverance and high consistency. As expected, paternal and maternal harsh discipline were each positively associated with aggressive behavior; the positive association between paternal harsh discipline and aggressive behavior was significant for adolescent boys with low levels of perseverance and consistency; moreover, the positive association between maternal harsh discipline and aggressive behavior was significantly stronger for adolescent boys with high levels of perseverance and consistency.

The first objective of this study was to explore the grit profiles of Chinese early adolescents. In line with our expectation, the finding revealed a three-profile solution of grit as optimal: profile 1 (low perseverance and low consistency), profile 2 (high perseverance and low consistency), and profile 3 (high perseverance and high consistency). Such a finding partially extends prior research on grit profiles in Chinese adolescents (Datu and Fong, 2018). Noteworthily, slightly distinct from their results, the current study adds a profile characterized by both low perseverance and low consistency. This may indicate that adolescents with low levels of determination to achieve long-term ambitions are also less likely to center on long-standing personal interests and targeted tasks.

Moreover, among these three profiles, most adolescents belonged to the profile characterized by both high perseverance and high consistency. One possible explanation is ascribed to Chinese culture, highlighting perseverance, self-discipline, and hard work (Lan et al., 2019a). In the meantime, the finding may be partially influenced by the nature of self-report measurements, which may be biased by social desirability (Podsakoff et al., 2003). In this regard, adolescents tend to report high levels of socially desired attributes (e.g., perseverance and consistency).

The second purpose was to examine the gender-specific patterns and the moderating role of grit profiles in the association between parental harsh discipline and aggressive behavior in Chinese early adolescents. In line with our expectations and prior research (Wang and Liu, 2014, 2018; Liu and Wang, 2018), the results showed that paternal and maternal harsh discipline were each positively associated with aggressive behavior. One possible interpretation is attributable to the social learning theory (Bandura, 1978). A primary caregiver’s responsiveness and the nature and timing of physical contact can act as “hidden” regulators of adolescents’ behavior. As such, parents who adopt high levels of power assertion and psychological aggression may trigger aggressive behavior of adolescents. In addition, the preliminary analysis showed that adolescents reported higher levels of paternal harsh discipline than maternal harsh discipline. This may be because fathers are more likely than mothers to be harsh and strict in terms of parenting practice and focus more on norm compliance (Shek, 2007; Li and Lamb, 2013).

Furthermore, the positive association between paternal harsh discipline and aggressive behavior was only significant for adolescent boys of the profile characterized by both low perseverance and low consistency. This finding can be interpreted by conventional Chinese parenting practice: fathers often take the main responsibility in disciplining their sons but leave the parenting of daughters to mothers (Ho, 1986). In this perspective, paternal harsh discipline may be more harmful in triggering boys’ aggressive behavior. Likewise, such an association is significantly heightened in the context of low levels of perseverance and consistency. This may be because adolescents who do not aspire to long-term goals are likely to have low self-control or self-regulatory ability. In this context, aggression toward others may allow adolescent boys to burst out their anger in response to paternal harsh discipline.

Surprisingly, the positive association between maternal harsh discipline and aggressive behavior was significantly stronger for adolescent boys of the profile characterized by both high perseverance and high consistency. Since fathers often take the primary responsibility in disciplining their sons, high and “additional” maternal harsh discipline to boys may further heighten their vulnerability. This may indicate that maternal harsh discipline to boys in the Chinese family context may intervene in their positive development pathway in early adolescence, which is highly discouraged. In this sense, boys may focus more on misbehaviors rather than on positive development. Moreover, in this context, adolescents with high perseverance and consistency demonstrated high aggressive tendencies. Such a finding is somewhat unexpected and counterintuitive. This is because grit, as a buffer, is generally assumed to be a positive personal attribute (Duckworth et al., 2007; Lan and Moscardino, 2019). One possible interpretation is in line with the indications of prior research (Anestis and Selby, 2015); they found that perseverance and consistency are related to impulsivity, which may enable boys to continuously focus on negative behavior, such as aggression, instead of achieving consistent personal positive growth.

In addition, the current research showed that the association between parental harsh discipline and aggressive behavior was not significant for girls, which was also regardless of the levels of perseverance and consistency. One possible explanation is that Chinese parents often mildly educate their daughters (Ho, 1986). As such, the low prevalence of aggressive behavior in girls may have resulted in failing to find a significant association. Such an interpretation is in line with the current findings; that is, girls exhibited lower levels of aggressive behavior than boys. Meanwhile, against our expectation, the grit profile characterized by high perseverance and low consistency did not show a robust buffering role against aggressive behavior (compared with the profile characterized by low perseverance and low consistency). This may indicate that the profile, highlighting high perseverance only, might not have a strong effect on decreasing aggressive behavior. One possible explanation is that consistency enables adolescents to focus on long-term aspirations, personal interests, and assigned tasks extensively. It may be, for example, that adolescents who do not concentrate on consistent interests and assigned tasks are more likely to show behavioral dysfunctions (e.g., attention deficits and hyperactivity), which in turn intensifies their aggressive tendencies toward others (Lan and Radin, 2019).

### Contribution to the Existing Literature

First, the current study extends prior research of parenting and aggression literature in early adolescence. During such a period, the tendency toward aggression potentially increases, and the ecological interaction between personal attributes and family context should be carefully considered to reduce adolescents’ misbehaviors.

Second, this study simultaneously investigates the roles of paternal and maternal harsh discipline in early adolescents’ aggressive behavior, in response to the emerging literature highlighting the role of fathers in adolescent development. Moreover, the gender differentiation effect in this association is investigated, which may increase the ecological validity of the findings, given that family interaction is regarded as a holistic unit. In this perspective, gender across both parents and their offspring may interactively influence adolescent development. In addition, according to the current findings, it facilitates our understanding of gender-differentiated parenting and its association with adolescent development in a specific cultural context. For example, both paternal and maternal harsh discipline may not reflect a risk for Chinese adolescent girls’ aggressive behavior.

Third, the current study contributes to the grit literature in several ways. In terms of the outcome, this study goes beyond achievement and performance issues by focusing on the behavioral variable. Moreover, we focus on the individual level of grit, but not on the variable level, to demonstrate how a latent profile analysis can generate empirically based grit profiles for Chinese early adolescents. The grit profiles revealed in this study indicate that perseverance and consistency may follow a similar developmental trend within each adolescent. This perspective may lead to additional theory development and practical insights for grit research. In addition, although grit is usually assumed to be a positive personal attribute, it may also contain a negative aspect in a specific context, which deserves further scientific attention and investigation. It is noteworthy that, for adolescent boys with high perseverance and consistency, maternal harsh discipline represents a heightened negative effect on aggressive behavior. This may indicate that grit, potentially linked to impulsivity, may have a double-edged-sword effect on adolescent development, especially for adolescent boys.

### Limitations and Implications

Although this study may contribute to the literature in several ways by documenting gender-specific patterns and the moderating roles of grit profiles in the association between parental harsh discipline and aggressive behavior among Chinese early adolescents, there are several limitations to acknowledge when interpreting the current findings. First, based on a cross-sectional design, this study does not have the power to establish causality among study variables. As indicated by prior research about the reciprocal relations between harsh discipline and children’s externalizing behavior (Wang and Liu, 2018), future studies should adopt a longitudinal design to ascertain the directionality of parental harsh discipline and aggressive behavior among Chinese early adolescents.

Second, the current study relies on self-report measurements, which are potentially influenced by social desirability and/or response bias (Podsakoff et al., 2003). As noted by prior research (Leung et al., 2006), externalizing behavior, such as aggressive behavior, is screened better by other informants (e.g., parent or teacher report) than self-report. Prospective future studies may obtain an external rater to confirm the current findings. Moreover, aggression is a multifaceted phenomenon, but the present study does not unpack different forms of aggressive behavior (e.g., physical aggression and verbal aggression; Björkqvist, 2018). For instance, prior research suggests that boys are more likely than girls to manifest episodes of online and offline hate, as boys may choose indirect ways to translate their anger into action (Pace et al., 2018). Future research is highly recommended to address the interplay of parents’ and adolescents’ gender, as well as grit profiles, in different forms of aggressive behavior. Such an approach can help researchers have a more comprehensive understanding of the linkage between parental harsh discipline and aggressive behavior.

Third, although a person-centered approach identifies patterns of many indicators rather than the relationship between these variables, the modest association between perseverance and consistency in this study may potentially influence the interpretation of the grit profiles. For instance, the current research does not find a “striking” distinction of perseverance and consistency within each adolescent. Researchers should keep this in mind when interpreting the present findings. From this perspective, further research may consider incorporating other dimensions of grit (e.g., adaptability to situations; Datu et al., 2017a) or broadening the domain specificity of grit (Cormier et al., 2019). This is because increasing the number of indicators may contribute to greater accuracy and certainty in defining the grit profiles. Likewise, using more indicators also improves convergence rates and reduces probability bias (Wurpts and Geiser, 2014).

Notwithstanding these limitations, the current study may have several theoretical and practical implications. With regard to theory, the present study enriches the social learning theory of aggression in a collective context (Bandura, 1978). When parents adopt harsh discipline to psychologically and behaviorally control their children’s behavior, their offspring may learn the effectiveness of hostile behavior through direct interaction with their parents, which could generalize to aggression in other social interaction contexts. Moreover, the current study suggests that aggressive behavior is regarded as a socioecological interaction between the individual and his or her family (Bronfenbrenner, 1979). Internal factors on the individual level interact with the family context, which then reinforces or inhibits their aggressive behavior. In order to develop truly effective intervention programs, it is imperative that researchers understand the complex ecological environment in which aggressive behavior takes place.

From an applied perspective, although Chinese cultural values highlight beating and scolding children as a reflection of parents’ love, the current findings indicate that parental harsh discipline represents a risk factor for early adolescents’ aggressive behavior. In this sense, family therapists and educational practitioners should work on minimizing the levels of parental harsh discipline and encouraging parents to change the traditional practice of disciplining their children. Moreover, in the context of high paternal harsh discipline, boys should be trained to improve in perseverance and consistency. For example, boys may be encouraged to set definite goals. When they encounter setbacks, consistent practice and attempts toward achieving these goals may enable them to overcome difficulties, in order to facilitate the development of perseverance and consistency (Lan and Moscardino, 2019). In addition, maternal harsh discipline to adolescent boys is highly discouraged, as it may potentially intervene in their positive development pathway. In this context, an intervention or prevention program aiming at facilitating their perseverance and consistency may have a negative effect on their behavioral development.

## Conclusion

By adopting a relatively large sample size and a person-centered approach, the current study demonstrates that both paternal and maternal harsh discipline present a risk for early adolescents’ aggressive behavior; however, such a detrimental effect is particularly harmful to adolescent boys. In the context of high paternal harsh discipline, adolescent boys should be trained to improve in perseverance and consistency, whereas, in the context of high maternal harsh discipline, it is suggested that adolescent boys de-emphasize perseverance and consistency.

## Data Availability Statement

The datasets generated for this study are available on request to the corresponding author.

## Ethics Statement

The studies involving human participants were reviewed and approved by the Wenzhou University. Written informed consent to participate in this study was provided by the participants’ legal guardian/next of kin.

## Author Contributions

GC conceived and drafted the manuscript. XL performed the statistical analyses and critically revised the manuscript. All authors read and approved the final draft of the manuscript.

## Conflict of Interest

The authors declare that the research was conducted in the absence of any commercial or financial relationships that could be construed as a potential conflict of interest.

## Footnotes

1 An interaction effect is the simultaneous effect of two or more independent variables on at least one dependent variable in which their joint effect is significantly greater (or significantly less) than the sum of the parts (see Lewis-Beck et al., 2004).

2 It is noteworthy that person-centered and variable-centered strategies represent complementary rather than competing approaches to research (Laursen and Hoff, 2006). The research question should dictate the selection of research strategies. As a basic distinction, a person-centered approach focuses on the individual but not on the variable; it identifies patterns of many indicators rather than the relationship between these variables (Bergman et al., 2006).
